# Nanobody-targeted photodynamic therapy for the treatment of feline oral carcinoma: a step towards translation to the veterinary clinic

**DOI:** 10.1515/nanoph-2021-0195

**Published:** 2021-08-02

**Authors:** Irati Beltrán Hernández, Guillaume C.M. Grinwis, Alessia Di Maggio, Paul M.P. van Bergen en Henegouwen, Wim E. Hennink, Erik Teske, Jan W. Hesselink, Sebastiaan A. van Nimwegen, Jan A. Mol, Sabrina Oliveira

**Affiliations:** Pharmaceutics, Department of Pharmaceutical Sciences, Faculty of Science, Utrecht University, 3584 CG Utrecht, the Netherlands; Cell Biology, Neurobiology and Biophysics, Department of Biology, Faculty of Science, Utrecht University, 3584 CH Utrecht, the Netherlands; Department of Biomedical Health Sciences, Faculty of Veterinary Medicine, Utrecht University, 3584 CL Utrecht, the Netherlands; Department of Clinical Sciences, Faculty of Veterinary Medicine, Utrecht University, 3584 CM Utrecht, the Netherlands

**Keywords:** comparative oncology, EGFR, feline cancer, nanobodies, photodynamic therapy

## Abstract

Nanobody-targeted photodynamic therapy (NB-PDT) has been developed as a potent and tumor-selective treatment, using nanobodies (NBs) to deliver a photosensitizer (PS) specifically to cancer cells. Upon local light application, reactive oxygen species are formed and consequent cell death occurs. NB-PDT has preclinically shown evident success and we next aim to treat cats with oral squamous cell carcinoma (OSCC), which has very limited therapeutic options and is regarded as a natural model of human head and neck SCC. Immunohistochemistry of feline OSCC tissue confirmed that the epidermal growth factor receptor (EGFR) is a relevant target with expression in cancer cells and not in the surrounding stroma. Three feline OSCC cell lines were employed together with a well-characterized human cancer cell line (HeLa), all with similar EGFR expression, and a low EGFR-expressing human cell line (MCF7), mirroring the EGFR expression level in the surrounding mucosal stroma. NB_A_ was identified as a NB binding human and feline EGFR with comparable high affinity. This NB was developed into NiBh, a NB-PS conjugate with high PS payload able to effectively kill feline OSCC and HeLa cell lines, after illumination. Importantly, the specificity of NB-PDT was confirmed in co-cultures where only the feline OSCC cells were killed while surrounding MCF7 cells were unaffected. Altogether, NiBh can be used for NB-PDT to treat feline OSCC and further advance NB-PDT towards the human clinic.

## 1 Introduction

NB-PDT has been developed since 2014 as a highly tumor-selective treatment [1], [2], [3], [4], [5], [6], [7], [8]. This strategy makes use of NBs to deliver a PS in the form of NB-PS conjugates specifically to tumor cells. NBs are the smallest naturally-derived antigen-binding fragments (∼16 kDa), which constitute the variable domain of heavy-chain antibodies occurring in camelids and cartilaginous fish [9]. Photosensitizers are light-activatable compounds that, upon accumulation in the tumor, can be excited by light of a particular wavelength locally applied at the tumor site, leading to the formation of reactive oxygen species and consequent cell death [10, 11]. In this respect, two levels of specificity, i.e. tumor targeting via NBs and local PS activation, make it possible to effectively kill cancer cells, while sparing surrounding healthy tissue.

NB-PDT has proven to be very specific in the *in vitro* setting for a wide range of membrane receptors, including EGFR [1, 2], human epidermal growth factor receptor 2 (HER2) [3], US28 G protein-coupled receptor (GPCR) [4] and the hepatocyte growth factor receptor (c-Met) [5]. *In vivo*, NB-PDT induces extensive damage to the tumor [2] and causes significant tumor regression after one single treatment session [3]. Permanent vascular effects including vasoconstriction, reduced perfusion and leakage have also been observed in the tumor area after NB-PDT [6]. Furthermore, the first indications of immunogenic cell death induced by NB-PDT have been recently reported [7], which suggests that antitumor immunity can be triggered [12]. These three antitumor mechanisms, i.e. direct tumor cell killing, tumor-associated vasculature effects and antitumor immunity, have been described for both conventional PDT [10] and antibody-targeted PDT [13]. Nevertheless, preclinical data indicate that NB-PDT offers advantages over these two PDT approaches.

Conventional PDT is nowadays used for many oncological indications, such as basal cell carcinoma, esophageal cancer and head and neck squamous cell carcinoma (HNSCC) [10, 14]. Despite good outcomes, the passive accumulation of the (usually hydrophobic) PS in the tumor generally leads to light application 24–72 h after injection, and the slow tissue clearance of the PS causes skin photosensitivity for days/weeks after treatment [10, 11]. Active targeting is achieved with antibody-targeted PDT, a strategy under evaluation in a phase III clinical trial for the treatment of HNSCC (NCT03769506) and recently approved in Japan to treat HNSCC patients [15]. Here, the anti EGFR antibody cetuximab is conjugated to the water-soluble PS IRDye700DX. However, the use of such a relatively large antibody with long circulation times still leaves several points of improvement, especially regarding tumor distribution of the conjugate and its slow clearance. The benefits of NB-PDT, which also uses IRDye700DX as PS, are the small size and high affinity of NBs which now guide the pharmacokinetics of the PS [2, 3]. These properties are responsible for the observed rapid and homogeneous accumulation of the conjugate in the tumor. Thus, illumination shortly after injection is feasible, which substantially improves the logistics of this treatment modality. Moreover, due to their small size, these conjugates are rapidly cleared from circulation by the kidneys, which potentially reduces the skin phototoxicity period after treatment. Altogether, the promise of NB-PDT is evident, and this prompts the next logical step of further translation to the clinic.

In view of clinical translation, EGFR-targeted NB-PDT has already been proven to be effective and selective using patient-derived HNSCC organoids and corresponding normal tissue organoids derived from the same patient [8]. Importantly, low/moderate EGFR levels were found on this patient cancer material, in comparison to common HNSCC cell lines. Although this led to a less potent effect of NB-PDT, tumor organoids were still killed, while normal organoids were unaffected. This highlights the importance of assessing treatment efficacy in the context of clinically relevant target expression. As the next step, we have directed ourselves to the possible application in oncological animal patients aiming to treat spontaneous tumors with high biological relevance; in particular, cats with OSCC. From all feline tumors, 10% of these occur in the oral cavity, being SCC the most abundant among these oral malignant tumors [16]. OSCC are fast-growing tumors and locally invasive, but they have a low metastatic potential. Limited success in the treatment of such tumors has been achieved so far by radiotherapy, chemotherapy, surgery or a combination of these [17, 18]. For most therapies, the medium survival time after diagnosis is around 3 months, where euthanasia is usually the only option due to the poor quality of life and limited options for a successful treatment [19]. The poor response of feline OSCC to current treatments, the superficial nature of these lesions, and infrequent metastasis account for the decision to investigate NB-PDT as a new treatment for these patients. In addition, feline OSCC shares similar pathogenesis, tumor biology and molecular markers with HNSCC [20], making it a very relevant choice to facilitate and accelerate translation to human patients. The fact that conventional PDT is already part of the arsenal to combat HNSCC in the clinic reinforces the choice to treat feline oral cancer with (targeted) PDT [10]. As a molecular target to be used by NB-PDT in feline OSCC, EGFR is a relevant choice due to its overexpression in HNSCC [21] as well as feline OSCC [22, 23]. Furthermore, the high homology of this protein between both species (92%, NCBI BLAST) broadens the possibilities to find cross-reactive NBs to the human and feline receptor to allow a smoother transition to the human clinic.

In this study, we describe the *in vitro* characterization of a species cross-reactive NB and its use for EGFR-targeted NB-PDT on a panel of feline OSCC cell lines, i.e. SCCF1, SCCF2, and SCCF3 cells. Bearing in mind the translation to the human clinic, a HeLa with clinically relevant target expression was taken along, with EGFR levels in the range of human head and neck cancers [8]. With the goal to demonstrate the specificity of this approach, a major advantage of NB-PDT, a human low EGFR-expressing cell line was included in the study (MCF7) as representation of the stroma cells of the normal oral mucosa [24, 25]. NB_A_ was identified from a previous panel of NBs selected against the extracellular domain of human EGFR described in [26], particularly from a screen of NBs that inhibit EGF binding to EGFR. The fact that moderate membrane EGFR levels are expected in the clinical setting [8, 25], together with the successful results obtained so far in clinical trials with antibody-PS conjugates bearing high PS to antibody ratios (NCT02422979) [27, 28], led us to develop a NB_A_-PS conjugate with high payload (average of 2.5 PS molecules per NB), named NiBh. This attractive conjugate maintained high affinity across species and target-specific potency when used for NB-PDT, highlighting its potential to treat cats with oral carcinoma and further advance the application of NB-PDT in the clinic.

## 2 Materials and methods

### 2.1 Cell lines and culture

The human cervical adenocarcinoma cell line HeLa and the human mammary adenocarcinoma cell line MCF7 were purchased from ATCC (ATCC CCL-2 and HTB-22). The feline oral squamous cell carcinoma cell lines SCCF1, SCCF2, and SCCF3 were kindly provided by Dr. Rosol (Ohio University). SCCF1 derives from a laryngeal SCC, SCCF2 from a bone-invasive gingival SCC, and SCCF3 from a lingual SCC [29, 30]. All cells were cultured in Dulbecco’s modified Eagle’s medium (DMEM) with high glucose and ultraglutamine 1 (Lonza, Basel, Switzerland) supplemented with 10% fetal bovine serum (FBS) (Sigma-Aldrich, Zwijndrecht, the Netherlands), 100 U/mL penicillin and 100 μg/mL streptomycin (Sigma-Aldrich). Cells were cultured at 37 °C and 5% CO_2_.

### 2.2 Nanobody and conjugation to fluorophore/photosensitizer

NB_A_ was produced and purified from the periplasmic fraction of *E. coli* as previously described [31].

For fluorescence detection, NB_A_ was conjugated to the fluorophore Alexa Fluor 647 NHS ester (Invitrogen, Carlsbad, CA, USA) following the manufacturer’s protocol, yielding a degree of conjugation (DOC), i.e. PS to NB ratio, of 1. Briefly, NB_A_ in PBS was incubated with the fluorophore for 2 h at room temperature, in a molar ratio of 1–4. Thereafter, free fluorophore was removed by size exclusion chromatography using three consecutive Zeba Spin Desalting Columns (Thermo Fisher Scientific, Perbio Science Nederland, Etten–Leur, the Netherlands).

For NB-PDT assays, NB_A_ was conjugated to the PS IRDye700DX (LI-COR, Biosciences, Lincoln, NE, USA). For this, the provider’s protocol was followed, controlling the conjugation conditions (i.e. time and temperature) to achieve NB-PS conjugates with DOC 0.5, 1, and 2.5, the latter referred to as NiBh. Free PS was removed using four consecutive Zeba Spin Desalting Columns. Further characterization of the NB-PS conjugates was performed as described in the Supplementary Materials.

### 2.3 Immunohistochemistry

Paraffin-embedded tissue blocks of feline OSCC were obtained from the archive of the Pathology division of the Veterinary Faculty at Utrecht University. Ten cases which contained OSCC as well as normal oral epithelium within the same tissue block were selected. For immunohistochemistry, 4 µm-thick tissue sections were mounted onto slides, deparaffinized and rehydrated. Antigen retrieval took place for 4 min at 37 °C with proteinase K (DAKO, Amstelveen, the Netherlands, cat no. S3020). Endogenous peroxidase was blocked with dual endogenous enzyme block (DAKO, S2003) for 10 min at room temperature and tissue was then blocked with PBS + 0.1% Tween (PBST) + 10% BSA for 1 h at room temperature. Tissue was incubated overnight at 4 °C with mouse anti-EGFR antibody (Thermo Fisher Scientific, MA5-13269) diluted 1:100 in PBST + 1% BSA. This primary antibody is a species cross-reactive antibody known to bind EGFR in dog, human, mouse, sheep, nonhuman primate and cat. Its detection was performed with the kit Envision + System-HRP anti-mouse (DAKO, K4401) for 30 min at room temperature. Staining was visualized by applying DAB chromogen (DAKO, K3468) and reaction stopped after 4 min. Slides were counterstained with hematoxylin. Canine skin was used as positive control tissue. As negative control, normal mouse IgG (Santa Cruz Biotechnology, Heidelberg, Germany, cat no. sc-2025) was used instead of the primary antibody. Immunoreactivity was evaluated as the product of percentage of positive tumor cells (1 ≤ 10%, 2 = 10–30%, 3 = 31–60%, or 4 ≥ 60%) and staining intensity (1 = low, 2 = moderate, and 3 = high), as in previous publications [22, 32]. Scores ≥2 were considered positive. To quantify staining intensity, ImageJ was used for deconvolution of the DAB color spectra and the optical density recorded in the regions of interest (tumor nests or basal layer of normal epithelium). Outcome was verified by an experienced veterinary pathologist (Guillaume C.M. Grinwis).

For immunohistochemistry with fluorescence detection, slides were subjected to the same steps as explained above. However, after incubation with the primary antibody, goat antimouse Alexa 555 (Invitrogen, A21424) was used as the secondary antibody (1:200 in PBST + 1% BSA) for 1 h at room temperature. Alternatively, to detect EGFR with NB_A_ instead, 10 nM of directly labeled NB_A_-Alexa 647 were incubated overnight at 4 °C. Slides were counterstained with DAPI (Roche, Basel, Switzerland). Images were taken with a confocal laser scanning microscope (Carl Zeiss Microscopy GmbH, Germany, LSM700) using a plan-apochromat 63x/1.40 Oil DIC and 20x/0.8 M27 objectives.

### 2.4 Binding assay

To assess the apparent binding affinity (K_D_) of NBs to the target in its natural conformation, EGFR-expressing human and feline cells were seeded in 96-well plates (10,000 cells per well) and incubated at 37 °C. The next day, cells were incubated for 2 h at 4 °C with a concentration range of NB (0.2–100 nM) in binding medium (DMEM without phenol red, supplemented with 25 mM HEPES and 1% BSA, pH 7.2). Unbound NB was washed off and cells fixed with 4% PFA (Merck, Haarlem, the Netherlands) for 10 min at room temperature. Bound NB was detected by incubating with rabbit anti VHH antibody (QVQ, Utrecht, the Netherlands, cat no. QE19) for 1 h at room temperature (1:1000 in PBS + 1% BSA) followed by goat anti rabbit IRDye800CW (LI-COR) for 1 h at room temperature (1:2000 in PBS + 1% BSA). Fluorescence at 800 nm was detected with an Odyssey infrared scanner (LI-COR). To assess the apparent binding affinity of the conjugates NB_A_-PS and NiBh, the same procedure was followed, but plates were directly scanned at 700 nm after washing off the unbound conjugate. Data was analyzed with GraphPad Prism software (GraphPad, La Jolla, CA, USA) and K_D_ values determined using a nonlinear fit with one-site specific binding. The K_D_, i.e. apparent dissociation constant, corresponds to the concentration of NB at which half of the total EGFR molecules are associated with NB.

The binding of NiBh was also assessed in the presence of EGF. For this, 10 nM of NiBh was incubated with the cells in the absence or presence of an equimolar concentration of EGF or a 10× molar excess of EGF (R&D Systems, Minneapolis, MN, USA, cat no. 236-EG).

### 2.5 Flow cytometry

Cells were added in a U-bottom 96 well-plate (10^5^ cells per well), washed once with PBS + 1% BSA and incubated for 45 min at 4 °C with 20 nM of mouse anti EGFR antibody. After washing, secondary antibody goat antimouse Alexa 488 diluted 1:200 was added and incubated with the cells for 30 min at 4 °C. Alternatively, to detect EGFR with NB_A_ instead, 40 nM of labeled NB_A_-Alexa 647 was incubated with the cells for 30 min at 4 °C. Unstained controls and samples stained with secondary antibody only were taken along for each cell line. Measurements were performed with a FACS Canto II (BD Biosciences, San Jose, CA, USA) and further analyzed with FlowLogic software (Inivai Technologies). The fluorescence intensity of both fluorophores was normalized to compare both in a single graph.

Resection material from two cats with OSCC was taken with informed owner consent at the University Clinic for Companion Animal Health (Utrecht University). To perform flow cytometry with primary feline OSCC tissue, the tissue was first cut into small fragments (∼5 mm^3^) and incubated for 30 min at 37 °C with 0.125% trypsin in Advanced DMEM/F12 (Life Technologies, Carlsbad, CA, USA) supplemented with 1× GlutaMAX, 100 U/mL penicillin–streptomycin and 10 mM HEPES (Life Technologies). The resulting cell suspension was filtered through a 100 μm cell strainer, followed by a 70 μm cell strainer. Red blood cells were lysed by incubating with a homemade RBC lysis buffer for 5 min on ice. Cell suspension was washed and subsequently stained for flow cytometry as described above for the cell lines.

### 2.6 Immunofluorescence on cells

Ten thousand cells per well were seeded in 16 wells Lab-Tek Chamber Slides (Thermo Fisher Scientific, 178599) and incubated at 37 °C. The next day, cells were washed with binding medium and costained with 20 nM mouse antiEGFR antibody and 40 nM NB_A_-Alexa 647 in binding medium. Incubation took place for 1.5 h at 4 °C and, subsequently, the cells were fixed with 4% PFA for 10 min at room temperature. The secondary antibody goat antimouse Alexa 488 (Invitrogen, A11029) was added for 1 h at room temperature (1:200 in PBS + 1% BSA). Cells were stained with DAPI and imaged with a confocal microscope.

### 2.7 Nanobody-targeted photodynamic therapy

NB-PDT was performed as previously described [1]. Briefly, cells were seeded in 96-well plates (10,000 cells/well) one day before the assay and incubated at 37 °C. The next day, cells were incubated with a concentration range of NB-PS conjugate (0.78–100 nM) for 30 min at 37 °C in PDT medium, i.e. DMEM without phenol red and L-glutamine (Lonza) supplemented with 10% FBS and antibiotics. Unbound conjugate was washed off and the plate was scanned with the Odyssey scanner at 700 nm to detect association of the conjugate with cells. Thereafter, cells were illuminated with 7 mW/cm^2^ for 59 min (25 J/cm^2^) using a 690 nm laser (Modulight ML7700, Tampere, Finland). Fluence rate was monitored with an Orion/PD optometer (Ophir Optronics, Jerusalem, Israel). After illumination, the plates were placed back at 37 °C.

NB-PDT was also performed with an excess of NB_A_, i.e. under competing conditions, with some minor adjustments of the above-mentioned protocol. Briefly, before adding NiBh (50 nM), cells were preincubated for 10 min at 37 °C with 10× or 50× molar excess of NB_A_. The unconjugated NB was present as well during the incubation with NiBh. In a different experiment, NB-PDT was performed in the presence of 25 mM or 50 mM sodium azide, as a quencher of singlet oxygen [33]. In this case, sodium azide was added to the cells only during the illumination time.

### 2.8 Cell viability and cell death after NB-PDT

One day after NB-PDT, cells were incubated with Alamar Blue reagent (Bio-Rad, Hercules, CA, USA) to assess viability, according to the protocol of the manufacturer. Fluorescence was measured with a FLUOstar Optima microplate reader (BMG Labtech, Ortenberg, Germany) and results expressed as cell viability in percentage relative to untreated cells. The median lethal dose (LD50), i.e. concentration of conjugate to achieve 50% of cell death, was determined using GraphPad Prism software with a log (inhibitor) vs normalized response fit.

Alternatively, 2 and 24 h after NB-PDT, live and dead cells were distinguished by staining with calcein AM (invitrogen) and propidium iodide (PI) (invitrogen) at a final dilution of 1:2000 and 1:1000, respectively, for 10 min at 37 °C. Cells were imaged with an EVOS microscope (Thermo Fisher Scientific) using transmitted light, a GFP cube for calcein AM, and an RFP cube for PI.

### 2.9 Co-cultures and specificity assays

SCCF2 and MCF7 cells were brought in suspension and labeled with the cell tracking dyes ViaFluor 405 and ViaFluor 488 (Biotium, Amsterdam, the Netherlands, cat no. 30068 and 30086), respectively, according to the provider’s protocol. Both cell lines were mixed in a ratio 1:1, seeded in 16 wells Lab-Tek Chamber Slides (Thermo Fisher Scientific) at a density of 10,000 cells per well and cultured at 37 °C. Cells were used the next day for further assays.

The cocultures were incubated with 50 nM of NB_A_-Alexa 647 in culture medium for 30 min at 37 °C. Alternatively, NB_A_-Alexa 647 was incubated for 1 h at 4 °C in binding medium. Thereafter, cells were fixed, stained with DAPI and imaged with a confocal microscope.

In a different assay, cocultures were treated with NB-PDT using 50 nM of NiBh. Cells were placed back in the incubator and, 2 h after illumination, cells were stained with PI (1:1000) and directly imaged with a confocal microscope.

## 3 Results

### 3.1 EGFR expression in feline oral squamous cell carcinoma and surrounding normal oral epithelium

**Figure 1:**
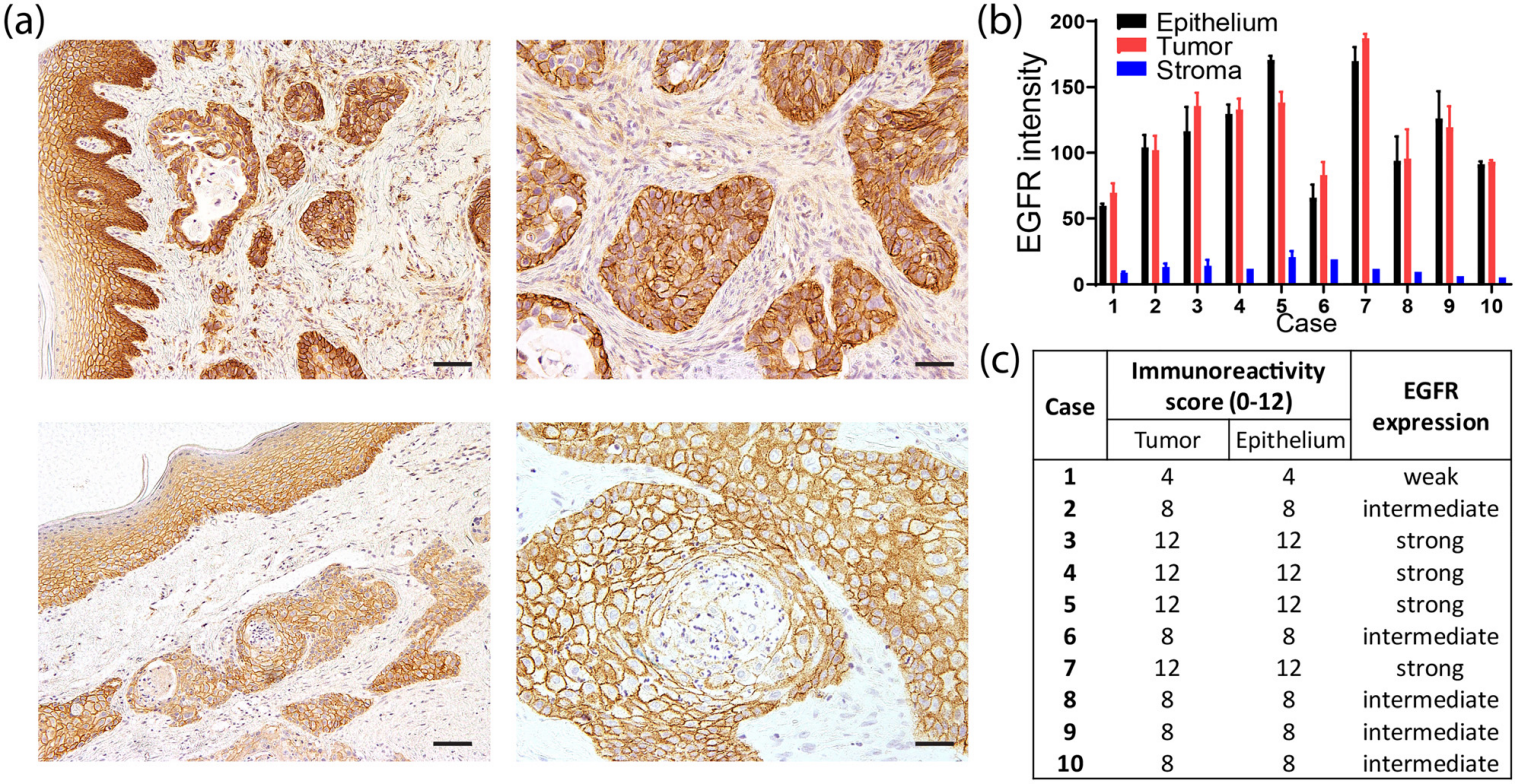
EGFR expression in feline OSCC and surrounding tissue. Immunohistochemistry was performed in 10 cases of OSCC with normal adjacent oral epithelium. (a) Representative images are shown of neoplastic nests compared to normal neighboring epithelium (left) and neoplastic nests surrounded by stroma (right), for two cases with strong (top), and intermediate (bottom) EGFR expression. Scale bar, 100 µm (left images) and 50 µm (right images). (b) Quantification of the EGFR signal in neoplastic nests (tumor), mucosal stroma (stroma), and basal layer of the adjacent oral epithelium (epithelium) per case. (c) Immunoreactivity score and classification of each case based on EGFR intensity calculated in B and % of positive tumor cells.

As EGFR has been described to be overexpressed in feline OSCC [22, 23], immunohistochemistry was performed on 10 cases of feline OSCC to investigate the expression of this protein in neoplastic cells and to elucidate its presence in cells of the surrounding normal oral mucosa (epithelium and stroma). EGFR presence, with predominant cell membrane localization, was detected in neoplastic cells forming nests and trabecula, and not found to be expressed in the surrounding stroma cells (Figure 1(a), right). On the other hand, membranous EGFR expression was also detected in the normal adjacent oral epithelium, more prominently in the basal cell layer and decreasing towards the outer, more differentiated epithelial layers (Figure 1(a), left). Interestingly, a similar pattern of differentiation and loss of EGFR expression was observed towards the core of large neoplastic nests which typically contain more differentiated neoplastic cells that can even show keratinization. For all 10 studied cases, EGFR positivity in the basal epithelial layer of preexisting normal epithelium was in the same range as in the neoplastic nests (Figure 1(b)). Accordingly, nine of the ten investigated cases expressed intermediate to high EGFR levels in the neoplastic nests, but EGFR was not overexpressed compared to the expression observed in the basal layer of the epithelium (Figure 1(c)). Altogether, these observations support that EGFR is a relevant target due to its expression in tumor cells and its absence in stroma.

### 3.2 Species cross-reactivity of NB_A_ to EGFR in human and feline cells

**Figure 2:**
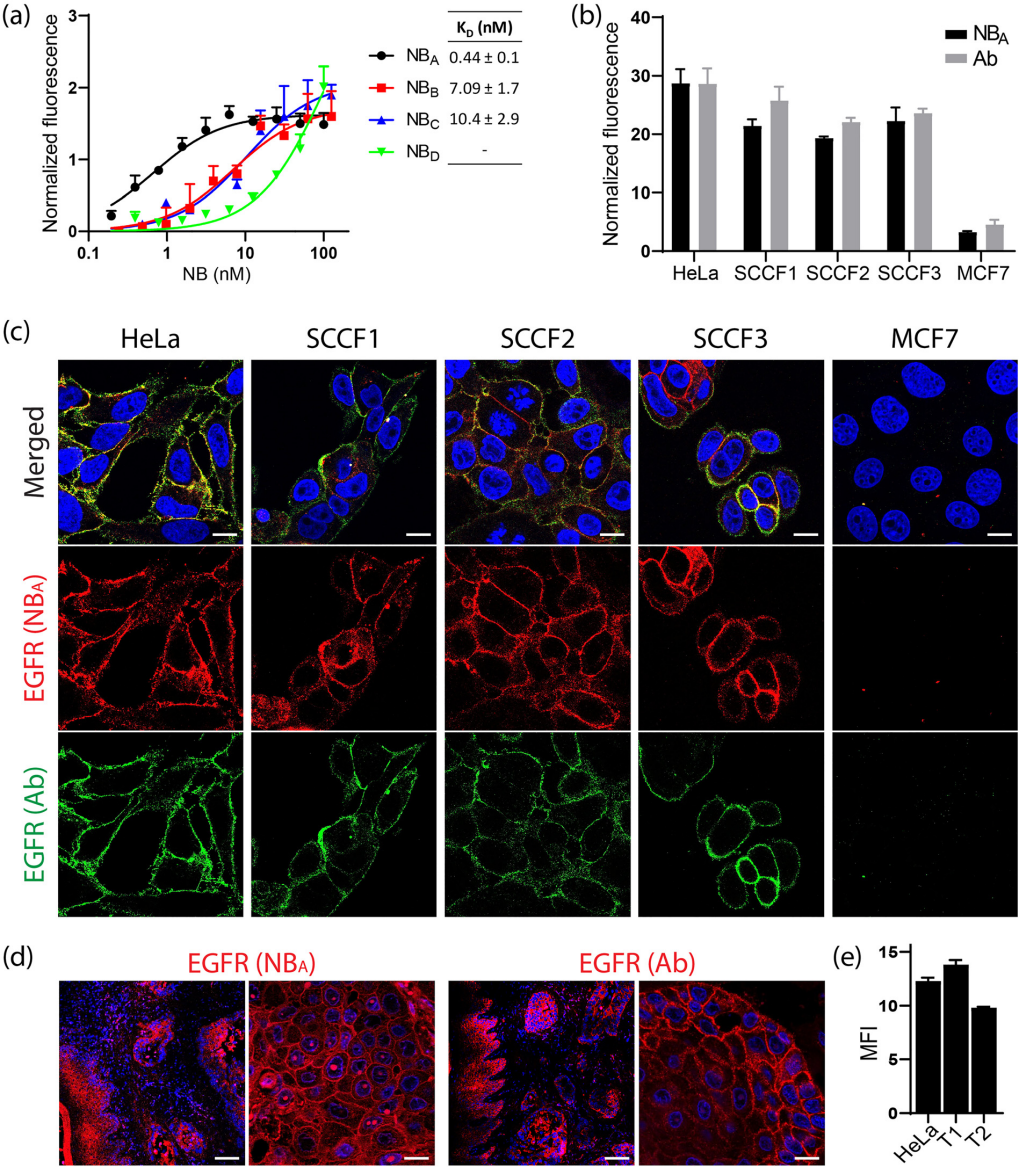
EGFR expression on human and feline cells and tissue detected by NB_A_. (a) A binding assay was performed with representative NBs, previously selected against human EGFR, using feline SCCF1 cells. The graph displays the normalized binding curves of several NBs to SCCF1 cells. The apparent binding affinities (*K*_D_) of the different NBs are shown. (b) and (c) Membrane EGFR was detected on three feline OSCC cell lines (SCCF1, SCCF2 and SCCF3) and two human cancer cell lines (HeLa and MCF7). (b) Membrane EGFR levels measured by flow cytometry using a commercial antiEGFR antibody or NB_A_-Alexa 647, expressed as median fluorescence intensity and normalized based on HeLa cells. (c) Confocal microscope images of each cell line co-stained for membrane EGFR using NB_A_-Alexa 647 (red, middle panel) and a commercial species cross-reactive antibody (green, bottom panel). Nuclei were stained with DAPI (blue). Merged images are shown on the top panels. Scale bar, 15 µm. (d) Feline OSCC tissue sections stained with NB_A_-Alexa 647 (left) or a commercial antiEGFR antibody (right). Images show an overview of the OSCC tissue including adjacent normal oral epithelium, and a close-up of the tumor cells. Scale bar, 100 µm (left images) and 15 µm (right images). (e) Membrane EGFR was detected on HeLa cells and primary tissue samples of two feline OSCC cases (T1 and T2). EGFR levels were measured by flow cytometry using a commercial antiEGFR antibody, expressed as median fluorescence intensity (MFI).

Having identified EGFR as a promising molecular target in feline oral carcinoma, we first confirmed the presence of membrane EGFR on three feline OSCC cell lines (SCCF1, SCCF2, and SCCF3), in comparison to two well-characterized human cancer cell lines (HeLa and MCF7) (Figure S1a). This was performed with flow cytometry using a commercial EGFR-targeting, species cross-reactive antibody. Thereafter, the binding affinity of a panel of NBs originally selected against human EGFR (with binding affinities, *K*_D_, <10 nM) was assessed on SCCF1 cells. From this NB panel, NB_A_ was identified as the most promising candidate due to its high binding affinity (*K*_D_ ∼ 0.44 nM) for feline cells (Figure 2(a)). To further investigate the species cross-reactivity of NB_A_ and its EGFR specificity, its binding to the three feline OSCC cell lines and the two human cancer cell lines was evaluated using flow cytometry and immunofluorescence. As a reference, the commercial EGFR-targeting antibody was used. NB_A_ was first conjugated to the fluorophore Alexa 647 (with binding affinity comparable to NB_A_, data not shown) to enable direct detection in the assays. The three feline OSCC cell lines had EGFR levels in the range of HeLa cells, while the fluorescence signal was minimal on the low EGFR-expressing MCF7 cells (Figure 2(b) and (c)). The same EGFR expression trend and pattern on the different cell lines was detected by the reference antibody, indicating the capability of NB_A_ to bind both human and feline EGFR (Figure 2(b) and (c)). Further supporting this, EGFR was also detected by NB_A_ (and the commercial antibody) on feline OSCC tissue (Figure 2(d)), predominantly at the basal epithelium and neoplastic nests. Interestingly, EGFR levels on primary feline OSCC tissue were in the same range as HeLa cells (Figure 2(e)) and, therefore, comparable to the feline OSCC cell lines used in this study. Quantification of the number of membrane EGFR molecules on the used human and feline cell lines indicated around 175,000 EGFR molecules per cell, while MCF7 cells (10,000 receptors per cell [34]) were below the detectable limit (Figure S1b).

### 3.3 Characterization of NiBh as an agent for EGFR-targeted PDT

**Figure 3:**
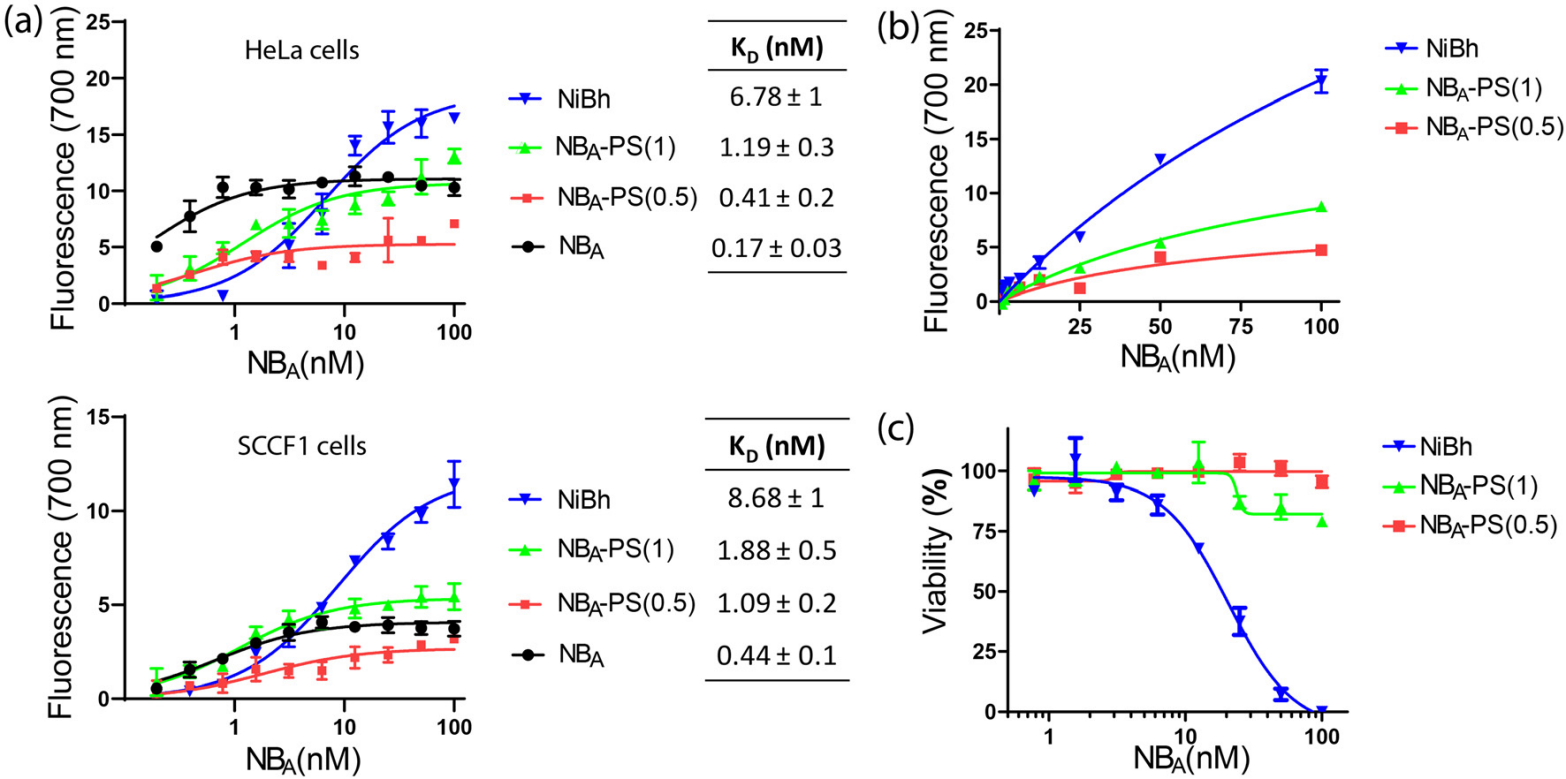
Binding affinity and potency of different NB_A_-PS conjugates. (a) A binding assay was performed with NB_A,_ NB_A_-PS(0.5), NB_A_-PS(1), and NiBh using HeLa (top) and SCCF1 cells (bottom). The graphs display the saturation binding curves of each targeting molecule per cell line. Apparent binding affinity (*K*_D)_ values of each NB and conjugate are shown. (b) To perform NB-PDT, a concentration range of NB_A_-PS(0.5), NB_A_-PS(1) or NiBh was incubated with SCCF2 cells for 30 min at 37 °C. The graph shows the PS signal on cells detected after washing off unbound conjugate, right before illumination. (c) The viability of the cells was assessed 24 h after illumination and expressed in percentage relative to nontreated cells.

NB_A_ was conjugated to the PS aiming to obtain conjugates with a different DOC, i.e. PS to NB ratios, in order to increase the PS density on tumor cells and, thus, cytotoxicity upon illumination. Accordingly, three conjugates were synthesized with 0.5, 1, or 2.5 PS molecules per NB molecule, named NB_A_-PS(0.5), NB_A_-PS(1), and NiBh, respectively. The conjugates were characterized in terms of purity by SDS-PAGE and the PS absorbance spectra was acquired, showing that the absorbance properties of the PS were not affected when conjugated to the NB, regardless of the DOC (Figure S2). Although the apparent binding affinities of the conjugates to both human and feline cells were slightly affected with an increasing degree of PS modification, the affinities still remained in the low nanomolar range (Figure 3(a)). Furthermore, the specificity of the conjugates for (feline) EGFR was verified by using EGFR knockdown SCCF1 cells, which resulted in a considerably reduced binding of the conjugate (Figure S3). When employing these conjugates for NB-PDT, it is evident that the use of NiBh resulted in the highest fluorescence (or density) of PS associated with the cells (Figure 3(b)) and, after illumination, NiBh was the only conjugate that induced significant cytotoxicity to the feline cells (Figure 3(c)).

**Figure 4:**
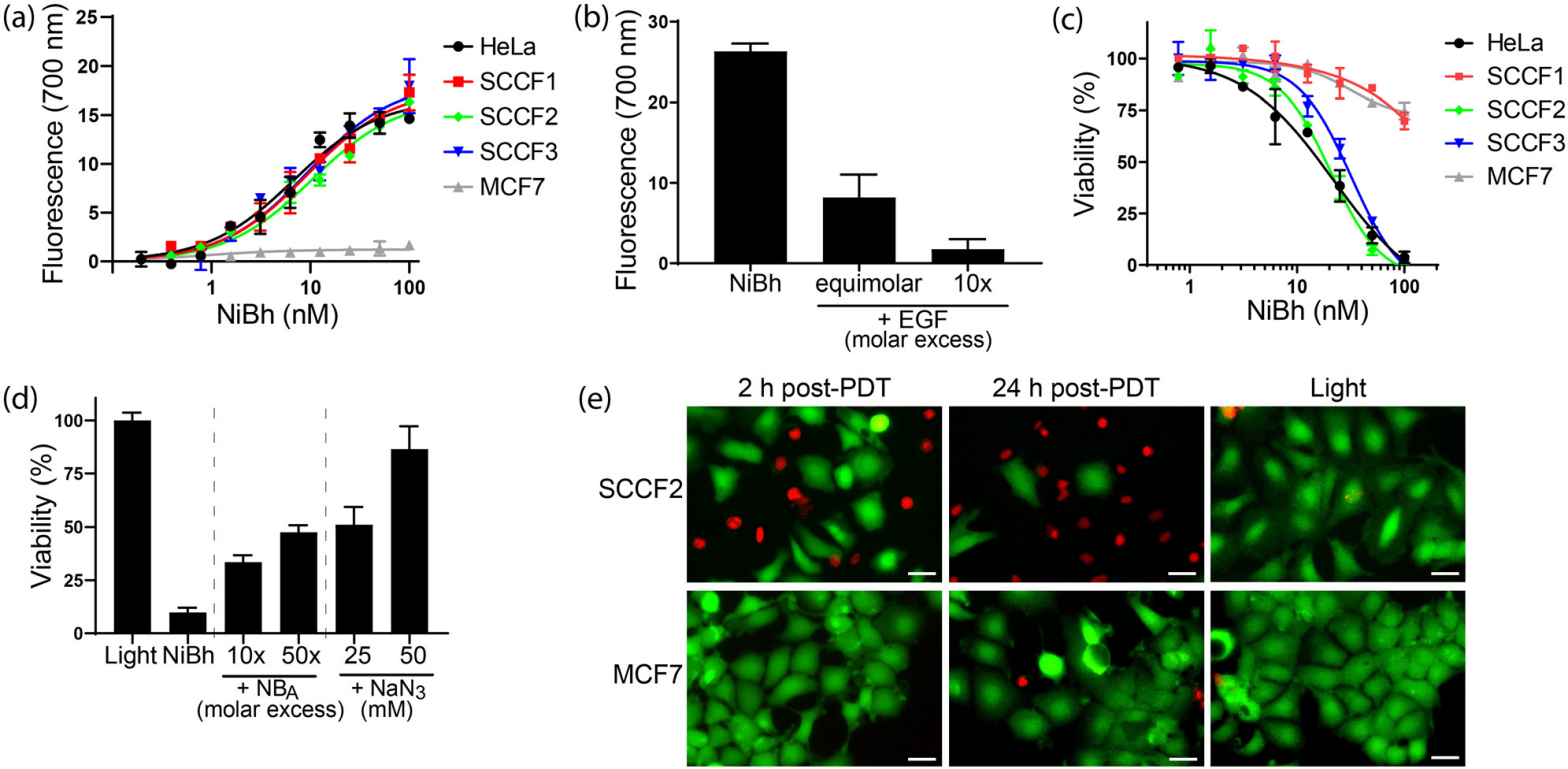
Specific binding and potency of NiBh. (a) A binding assay was performed with NiBh using the panel of human and feline cell lines (HeLa, SCCF1, SCCF2, SCCF3, and MCF7 cells). The graph displays the saturation binding curves of NiBh to each cell line from which K_D_ values were calculated. (b) Binding of NiBh (10 nM) to SCCF2 cells in the absence or presence of equimolar concentration and 10× molar excess of EGF. The fluorescence corresponding to bound NiBh under each condition is displayed in the graph. (c) NB-PDT using NiBh was performed on the panel of cell lines and, one day later, viability was assessed. The graph shows viability curves for each cell line, in percentage relative to untreated cells. (d) NB-PDT (50 nM NiBh) was performed on SCCF2 cells in the absence or presence of a molar excess of unconjugated NB_A_. NB-PDT was also performed in the presence of sodium azide (NaN_3_). A control consisting of cells exposed to light, but no conjugate, was included. Cell viability was assessed the next day and represented as percentage relative to untreated cells. (e) NB-PDT (50 nM NiBh) was performed on SCCF2 or MCF7 cells and cells stained with calcein and propidium iodide to visualize live (green) and dead (red) cells, respectively. A control in which light was applied, but no conjugate, was taken along. Images were taken 2 and 24 h after NB-PDT. Scale bar, 20 µm.

NiBh, the NB_A_-PS conjugate with highest payload, was selected as the best agent for EGFR-targeting NB-PDT on feline OSCC cells. The special feature of this conjugate is its high binding affinity to both human and feline cells (K_D_ ∼ 7–10 nM) while having DOC 2.5 (Figure 4(a)). In addition, the specific binding of NiBh to feline EGFR was maintained, as evidenced by a reduced NiBh binding in the presence of EGF in a concentration dependent manner (Figure 4(b)). The potency of NiBh as an agent for NB-PDT was evaluated using the panel of human and feline cell lines (Figure 4(c)). HeLa, SCCF2, and SCCF3 cells were effectively killed after treatment with comparable low nanomolar LD50 values (HeLa, 15.9 ± 3.7 nM; SCCF2, 17.8 ± 2.2 nM; SCCF3, 26 ± 3.5 nM). In line with the very low EGFR expression on MCF7 cells, these cells were only slightly affected at the highest concentrations of the conjugate. On the other hand, the use of NiBh for NB-PDT on SCCF1 cells resulted in only slight cytotoxicity, contrary to the other cell lines with comparable EGFR expression. To further investigate whether the induced cytotoxicity relies on the specific binding of NiBh to EGFR, NB-PDT was performed in the presence of an excess of unconjugated NB_A_. Accordingly, the cytotoxicity decreased under these competing conditions (Figure 4(d)). Moreover, the NB-PDT effect could be inhibited in the presence of sodium azide (NaN_3_), a singlet oxygen quencher [33] (Figure 4(d)). To visualize the distinct NB-PDT effect on moderate and low EGFR-expressing cells, SCCF2, and MCF7 cells were stained with fluorescent dyes denoting live/dead cells after NB-PDT. Already early after NB-PDT (2 h), dead SCCF2 cells were clearly distinguishable and their number increased over time, while only a very small number of dead MCF7 cells could be detected one day after treatment (Figure 4(e)).

### 3.4 Specific tumor cell killing mediated by NB-PDT using NiBh

**Figure 5:**
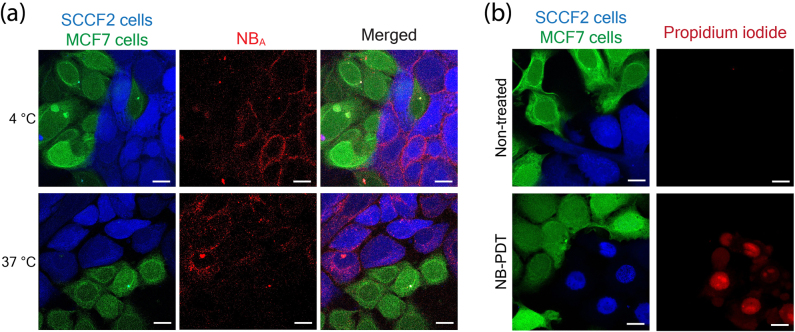
Specificity of NB_A_ and NiBh in cocultures. SCCF2 cells were labeled with ViaFluor 405 (blue), MCF7 cells with ViaFluor 488 (green), and co-cultured in a 1:1 ratio. (a) Confocal images of cells incubated with 50 nM of NB_A_-Alexa 647 (red) for 1 h at 4 °C (top) or 30 min at 37 °C (bottom). Scale bar, 15 µm. (b) Confocal images of cells 2 h after NB-PDT with 50 nM of NiBh. Cells were stained with propidium iodide to distinguish dead cells (red). Scale bar, 15 µm.

To investigate the specificity of NiBh for NB-PDT in a representative and biologically relevant setting, co-cultures were set up consisting of feline OSCC cells (SCCF2), as neoplastic nests, and low EGFR-expressing cells (MCF7) mimicking the surrounding stroma of the oral mucosa. First, NB_A_-Alexa 647 was employed to visualize its differential binding (4 °C) to both cell lines. As anticipated, NB_A_ was found on the membrane of the feline carcinoma cells, while the detected signal was minimal on the surrounding low EGFR-expressing cells (Figure 5(a), top). The next step was to address the accumulation of NB_A_-Alexa 647 in cells after an incubation time mimicking the incubation with NB-PS conjugate used for the NB-PDT studies (30 min at 37 °C). This revealed a predominant accumulation of the NB in the feline tumor cells both membrane-bound and internalized (Figure 5(a), bottom). Lastly, NB-PDT using NiBh was performed on the cocultures and dead cells were visualized via PI staining. Most feline neoplastic cells were killed shortly after treatment (2 h), whereas neighboring low EGFR-expressing cells were left unaffected (Figure 5(b)).

## 4 Discussion

NB-PDT has emerged as a potent and selective treatment modality for cancer able to effectively kill cancer cells while sparing surrounding healthy tissue. In particular, the use of NB-PDT to treat HNSCC has been demonstrated both *in vitro* [1] and in the preclinical setting [2, 6] with great success. Furthermore, in view of clinical translation, NB-PDT has shown its value using patient-derived HNSCC organoids [8]. This prompted us to advance NB-PDT further and the next logical step was to bring this treatment closer to the human clinic. For this, we first opted to develop NB-PDT for treating companion animals suffering from spontaneous tumors in the veterinary clinic. In particular, we aim to treat cats with OSCC, a tumor type greatly resembling human HNSCC, with no current effective therapeutic options. In the present study, we describe *in vitro* work that paves the way to the *in vivo* application of NB-PDT in cats with OSCC. EGFR was selected as a target for feline OSCC and, accordingly, NB_A_ was characterized as an EGFR-targeting NB binding both the human and the feline protein. Next, NB_A_ was conjugated to IRDye700DX to yield NiBh, a NB-PS conjugate with high payload (average of 2.5 molecules of PS per NB molecule) which retains high binding affinity to both species. NiBh was subsequently investigated for NB-PDT to specifically kill feline OSCC cells with EGFR levels comparable to those of neoplastic cells *in vivo*, whereas neighboring low EGFR-expressing cells were left unharmed.

Expression of EGFR in feline OSCC has been reported and suggested as therapeutic target [22, 23]. Looper et al. found moderate/high EGFR expression in 8 of 13 cases [22], while we found it in 9 out of 10 cases (Figure 1). Despite being a small subset of samples in both instances, it is clear that EGFR is present in feline OSCC to a significant extent. EGFR was confined to the cancer cells arranged in neoplastic nests and absent in the surrounding stroma, which, in the context of NB-PDT, will most likely result in no damage to the structural component of the oral mucosa. Nonetheless, we compared EGFR expression in the adjacent normal oral epithelium and observed that the intensity in the basal layer of this epithelium was similar to the neoplastic cells, in line with the observations in HNSCC [25, 35]. Intermediate and high EGFR levels have been reported in feline normal oral mucosa/tongue [22], but never compared side by side with OSCC tissue. These comparable EGFR levels would render the epithelial cells of the non-lesional mucosa susceptible to EGFR-targeted PDT, but this is not regarded as a concern due to the local nature of PDT and rapid regeneration of the feline oral mucosa [36]. Furthermore, conventional PDT has already been applied in cats for the treatment of SCC and only transient and acceptable local adverse effects were observed with this nontargeted approach [37], [38], [39], [40], thus NB-PDT would further minimize these undesired effects.

We consider EGFR the best target with regard to NB-PDT for treatment of feline OSCC. Next to EGFR, only soluble molecules (e.g. VEGF and COX-2) have been reported as highly expressed in feline OSCC [20], which cannot be utilized for NB-PDT. More is known about markers in human HNSCC, where HER2 and HER3 have gained attention. Nevertheless, while EGFR is expressed in more than 90% of HNSCC and is a clinically validated therapeutic target in HNSCC [41], the other two family members are less commonly expressed [42] and its targeting has resulted in modest clinical success so far [43]. Novel biomarkers for HNSCC that could be used for NB-PDT are emerging, such as CD44, c-Met and PD-L1 [41, 44], but further studies are warranted and their presence in feline OSCC is unknown.

It has been described that HNSCC cell lines express higher EGFR levels than what is generally found in the cancer tissue [25]. Therefore, our aim was to ensure that we work with cells expressing clinically relevant EGFR levels, to assess the extent of cytotoxicity induced by NB-PDT that can be expected in the clinic. All feline OSCC cell lines presented membrane EGFR levels comparable to HeLa cells. This is in agreement with our results obtained with patient-derived HNSCC organoids, for which EGFR expression levels were also in the range of HeLa cells [8]. With the idea of clinical relevance in mind, MCF7 cells served as very low EGFR-expressing cells [24], representing the adjacent stroma present around the neoplastic nests in feline OSCC. The first proof of the ability of NB_A_ (originally selected against human EGFR) to recognize human and feline EGFR was denoting these differences in EGFR expression between the cell lines, with a comparable trend to a commercial EGFR-targeting, species cross-reactive antibody (Figure 2). This makes NB_A_ the first reported NB to bind a feline target, thus joining the small group of NBs developed against targets in companion animals, next to the canine HER2-targeting NBs [45].

In order to use NB_A_ for NB-PDT, the critical step of conjugation to the PS was carefully considered. NB-PDT makes use of NB-PS conjugates with good pharmacokinetics for PDT due to the small size of NBs. At the same time, however, this small size allows for only a modest amount of payload (0.5–1.5 molecules per NB) without affecting the overall binding affinity of the NB [46]. On the other hand, one antibody can easily carry 3–4 drug molecules [27, 47]. So far, NB-PDT has proven potent using monomeric and dimeric NB-PS conjugates with DOC 0.5–1.5, using moderate and high expressing cell lines [2, 3]. Dimeric NBs (e.g. internalizing biparatopic NBs) facilitate the incorporation of a higher number of PS molecules and have proven to be very potent *in vitro* [1], but their penetration in a solid tumor is limited by their larger molecular size [2, 48, 49], and thus not the first choice for the clinic in the context of NB-PDT. To ensure a potent NB-PDT effect in a spontaneous tumor and because clinical success has been obtained with antibody-PS conjugates with high DOC [27, 28], we developed NB_A_ into NiBh, a monomeric NB-PS conjugate with high DOC (2.5). NiBh is, to our knowledge, the first functional monomeric NB with such a high number of payload (e.g. fluorophore or drug) [46].

Importantly, NiBh retained high affinity to both human and feline EGFR, even with high DOC (Figure 3(a)). This is, so far, a unique feature of NB_A_ since other species cross-reactive NBs lack this comparable affinity across species [45, 50]. NiBh could effectively kill the different cell lines with clinically relevant EGFR expression (LD50 in low nanomolar range), while cytotoxicity was not induced with NB-PS conjugates with lower DOC (Figures 3(c) and 4(c)). This can be explained by the concept of the threshold dose, which describes that cellular damage is induced only above a certain concentration of reactive oxygen species [51]. The NB-PDT effect induced by NiBh was further characterized as dependent on the binding to EGFR and mediated by the formation of singlet oxygen (Figure 3(d)), as reported for antibody-targeted PDT employing the same PS [52]. The induced cytotoxicity occurred in an EGFR-dependent manner, a correlation that has been described for NB-PDT with other conjugates [1, 8]. Only SCCF1 cells did not behave as anticipated, since only a moderate NB-PDT effect was observed, while a more pronounced effect was expected based on the EGFR levels of this cell line. Differences in PDT-induced cytotoxicity have been attributed to variations in the level of antioxidant molecules and enzymes expressed by cancer cells, but this was proved not to be the case for the feline OSCC cell lines (Figure S4). Many other cellular mechanisms play a role in the degree of PDT cytotoxicity [53] and further investigation would be needed to clarify the observed differences in the response. Of note, it is clear that the cytotoxicity of EGFR-targeted PDT is independent of EGFR downstream mutations and the presence of membrane EGFR is the main driver. Another important aspect of NB-PDT that differentiates it from conventional PDT is its ability to leave illuminated surrounding normal tissues unharmed. This was indeed the case with NiBh in cocultures representing feline OSCC surrounded by low EGFR-expressing stroma cells (Figure 5).

Conventional PDT has already been employed in the veterinary clinic to treat various cancers, such as oral, bladder and prostate carcinomas in dogs and cats [54], [55], [56], [57]. Targeted PDT, however, has not yet been applied in companion animals. Thus far, antibody-targeted PDT has shown efficacy in a xenograft mouse model of canine bladder cancer [58], but follow-up studies have not yet been reported. For the treatment of cutaneous, nasal, and facial SCC in cats, conventional PDT has yielded variable responses depending on stage and tumor location [37], [38], [39], [40]; but, as far as we know, there are no reports concerning treatment of OSCC. We believe that NB-PDT differentiates itself from the efforts made so far with conventional PDT to treat feline SCC. Potential benefits associated to NB-PDT are a more rapid and homogeneous tumor accumulation of the PS, milder local effects due to selective cell killing, increased light penetration through tissues due to the near infrared wavelengths required to activate the PS IRDye700DX, combination of imaging and treatment due to the versatility of IRDye700DX to act as a fluorophore, minimal photosensitivity after treatment, and treatment protocol performed within one day. These are all strong points to support the use of NiBh to treat feline OSCC in the clinic and, consequently, our next efforts will be pointed in this direction. In particular, we will seek to apply NB-PDT as an experimental treatment in client-owned cats with OSCC, having confirmed moderate/high EGFR expression.

In conclusion, NiBh can be used *in vitro* to kill feline OSCC cells that express clinically relevant EGFR levels, while sparing surrounding low expressing cells. Therefore, this study presents the use of NiBh for NB-PDT as an attractive therapeutic modality for the treatment of feline OSCC in the veterinary clinic. The species cross-reactivity of NiBh and the similarities of feline OSCC with HNSCC make NB-PDT a promising treatment option for human patients and position the translation of NB-PDT to the human clinic one step closer. In the long term, NiBh could be employed against other EGFR-expressing cancers and with a potential use in other species as well.

## Supplementary Material

Supplementary Material Details
