# Supplementary material for: Nanobody-targeted photodynamic therapy for the treatment of feline oral carcinoma: a step towards translation to the veterinary clinic
Source: Nanophotonics. 2021 Aug 2;10(12):3075–87. doi: 10.1515/nanoph-2021-0195 (PMC9646246; doi:10.1515/nanoph-2021-0195)
Supplement: Supplementary file 1 — Supplementary Material Details [file j_nanoph-2021-0195_suppl.pdf]

Nanobody-targeted photodynamic therapy for the treatment of feline oral carcinoma: a step towards translation to the veterinary clinic

Irati Beltrán Hernández[1,2], Guillaume C.M. Grinwis[3], Alessia Di Maggio[2], Paul M.P. van Bergen en Henegouwen[2], Wim E. Hennink[1], Erik Teske[4], Jan W. Hesselink[4], Sebastiaan A. van Nimwegen[4], Jan A. Mol[4] and Sabrina Oliveira[1,2,*]

1 Pharmaceutics, Department of Pharmaceutical Sciences, Faculty of Science, Utrecht University, 3584 CG Utrecht, the Netherlands

2 Cell Biology, Neurobiology and Biophysics, Department of Biology, Faculty of Science, Utrecht University, 3584 CH Utrecht, the Netherlands

3 Department of Pathobiology, Faculty of Veterinary Medicine, Utrecht University, 3584 CL Utrecht, the Netherlands

4 Department of Clinical Sciences of Companion Animals, Faculty of Veterinary Medicine, Utrecht University, 3584 CM Utrecht, the Netherlands

*****Correspondence: s.oliveira@uu.nl; Tel.: +31-63-410-3460

Supplementary information

Methods

Quantification of membrane EGFR molecules per cell

Cells were seeded in a 96-well plate (10,000 cells/well) and incubated at 37 °C. Two days later, cells were incubated for 2 hours at 4 °C with a concentration range of NB_A-IRDye800CW (conjugated as in [1]) in binding medium (DMEM without phenol red, supplemented with 25 mM HEPES and 1 % BSA, pH 7.2). Unbound NB was washed off and the bound fraction was brought in suspension by incubating the cells with acid wash buffer (0.2 M glycine-HCl and 150 mM NaCl, pH 2.3) twice, for 6 and 3 minutes. Both fractions were transferred to a new plate, neutralized (1 M Tris-HCl, pH 9), and scanned with an Odyssey infrared scanner (LI-COR). Data was analyzed with GraphPad Prism software to determine the Bmax, i.e. total receptor density. This value was interpolated in a titration curve made with NB_A-IRDye800CW, to obtain molecules of nanobody per well using the Avogadro constant. Assuming binding of one nanobody per EGFR molecule and counting the cells per well, the number of EGFR molecules per cell was calculated. In this assay, A431 cells were included as a known EGFR-overexpressing cell line (1–2 million receptors per cell [2]) to confirm the setup of the assay.

Characterization of NB–PS conjugates

The purity and integrity of the different NB-PS conjugates and unconjugated NB were assessed by size separation via 15 % SDS-PAGE. The gel was scanned at 700 nm with an Odyssey scanner (LI-COR) to detect the PS fluorescence. Afterwards, total protein was stained with Page Blue (Thermo Fisher Scientific) and detected again with the infrared scanner. In addition, the absorption spectra (230 – 750 nm) of each NB and conjugate was measured with a NanoDrop spectrophotometer (NanoDrop Technologies).

EGFR knockdown and binding assay

SCCF1 cells were seeded in a 96-well plate (10,000 cells/well) and incubated at 37 °C. The next day, cells were transfected with EGFR Stealth siRNA (Thermo Fisher, EGFR-3236) following the standard protocol for Lipofectamine 2000 (Invitrogen, 11668-027). Non-transfected cells were taken along, and non-targeting siRNA (Qiagen, SI03650318) was employed as negative control. After 48 hours, cells were collected and membrane EGFR stained to verify the knockdown. Staining was performed by incubation for 45 minutes at 4 °C with mouse anti-EGFR antibody (ThermoFisher, MA5-13269) diluted 1:75, followed by goat anti-mouse Alexa 488 (Invitrogen, A11029) for 30 minutes at 4 °C (diluted 1:200). HeLa cells as reference cell line and unstained controls were taken along. Measurements were performed with a FACS Canto II (BD) and further analyzed with FlowLogic software (Inivai Technologies).

The binding of NB_A-PS(1) on EGFR knockdown SCCF1 cells was assessed. Two days before the assay, SCCF1 cells were left untreated or transfected with EGFR gene siRNA as explained above. After 48 hours, cells were incubated for 2 hours at 4 °C with a concentration range of NB_A-PS(1) in binding medium (DMEM without phenol red, supplemented with 25 mM HEPES and 1 % BSA, pH 7.2). Unbound conjugate was washed off and PS fluorescence detected on cells with an Odyssey scanner (LI-COR) at 700 nm.

Antioxidant capacity

Cells were collected (200,000 cells per cell line), pelleted and resuspended in MQ water with 0.5 % Triton X-100 (Sigma-Aldrich). After 10 minutes on ice, cell debris was removed by centrifugation and supernatant used for the assay. Samples were processed as indicated by the manufacturer with a Total Antioxidant Capacity Assay Kit (Sigma-Aldrich, #MAK187), without using the optional Protein Mask reagent. Absorbance at 550 nm was measured with a FLUOstar Optima microplate reader (BMG Labtech) and data analyzed with GraphPad Prism software to calculate concentration of antioxidant in each sample.

Figures

(a)

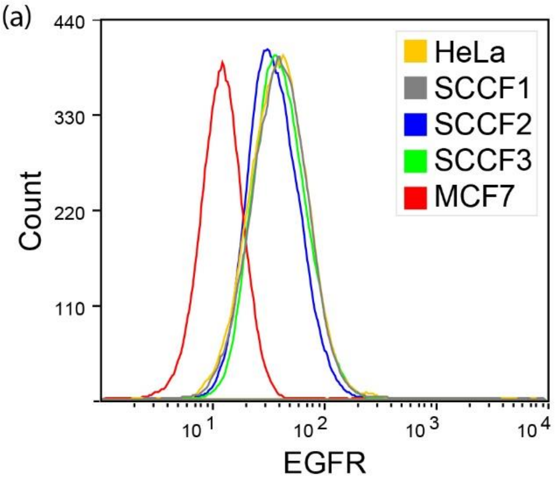

(b)

Cell line	EGFR molecules per cell (x 10^3)
A431	1,650 ± 70
HeLa	150 ± 85
SCCF1	175 ± 60
SCCF2	190 ± 55
SCCF3	195 ± 60
MCF7	-

Figure S1. Detection of membrane EGFR on several human and feline cell lines. (a) EGFR was detected by flow cytometry on three feline OSCC cell lines (SCCF1, SCCF2 and SCCF3) and two human cancer cell lines (HeLa and MCF7) using a commercial anti-EGFR, species cross-reactive antibody. Histogram shows the fluorescence intensity corresponding to EGFR for each cell line. (b) The number of membrane EGFR molecules per cell was quantified based on the saturation binding of NB_A-IRDye800CW. Average results are shown, calculated from at least three independent experiments. A431 cells were included as a known EGFR-overexpressing cell line. MCF7 cells are reported to have 10,000 EGF receptors per cell [3], but such low numbers are below the detection limit of the assay.

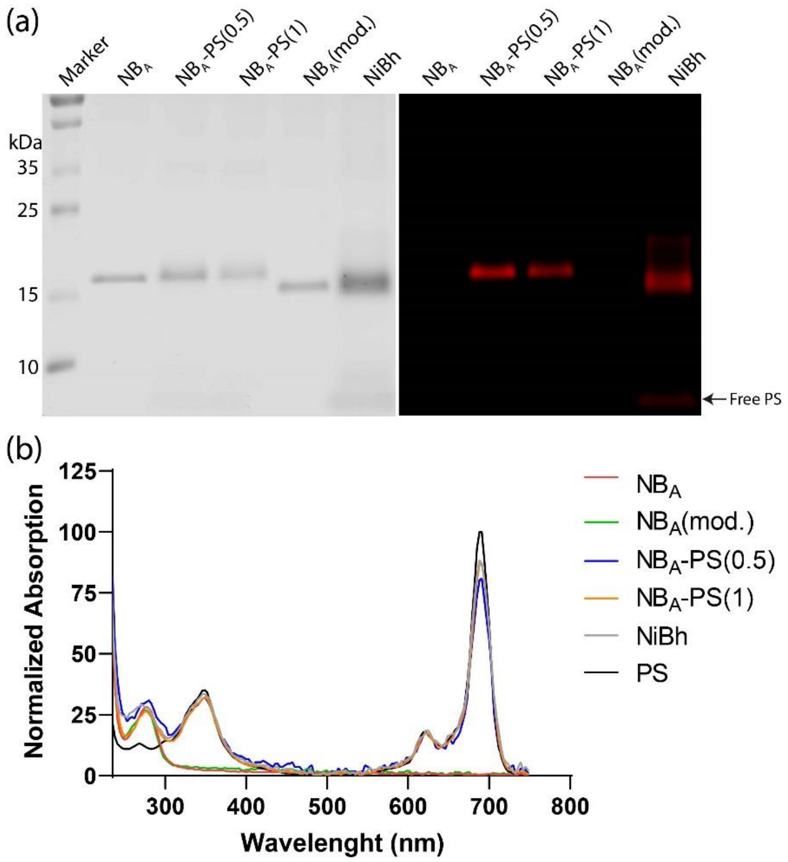

Figure S2. Characterization of the different conjugates. NB_A was conjugated to the photosensitizer IRDye700DX achieving DOC 0.5 (NB_A-PS(0.5)) or DOC 1 (NB_A-PS(1)). The sequence of NB_A was slightly modified (NB_A(mod.)) to enable yielding DOC 2.5 (NiBh). (a) SDS-PAGE of unconjugated and conjugated NB_A. The right gel shows the PS signal of each protein, while the left gel displays total protein. Arrow points to the free PS remaining in the conjugates, which is in the range of 10% and lacks cytotoxicity on its own [4]. (b) The absorption spectra of NB_A, every conjugate and the photosensitizer (PS) alone was measured in the range 230 – 750 nm.

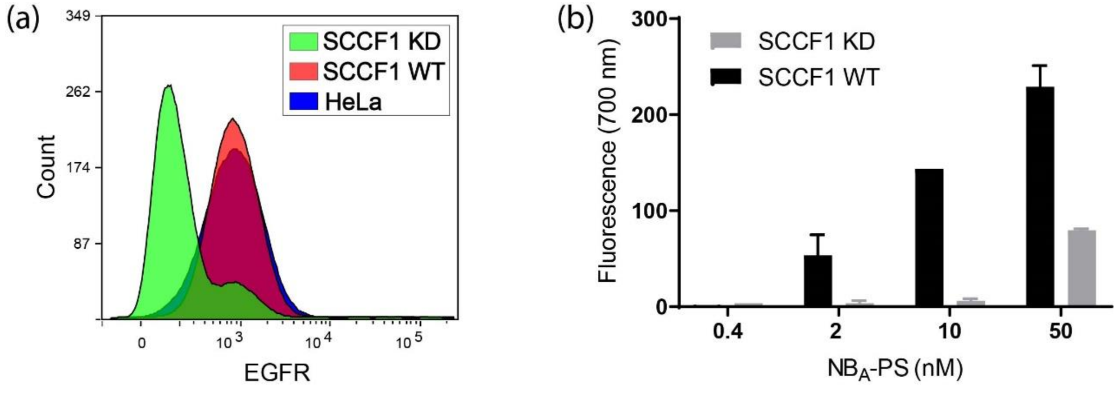

Figure S3. Binding of NB_A-PS to EGFR knockdown feline cells. (a) EGFR was knocked down on SCCF1 cells and this was confirmed by detecting membrane EGFR levels on wild type and knockdown SCCF1 cells, SCCF1 WT and SCCF1 KD respectively, using

a commercial anti-EGFR, species cross-reactive antibody. HeLa cells were taken along as a reference of moderate EGFR expression. Histogram shows the fluorescence corresponding to EGFR for each cell line. About 30 % of the cells retained EGFR expression comparable to the wild type cell line. (b) The binding of NB_A-PS(1) was assessed on wild type and knock down SCCF1 cells. The fluorescence corresponding to the bound conjugate to each cell line is shown in the graph.

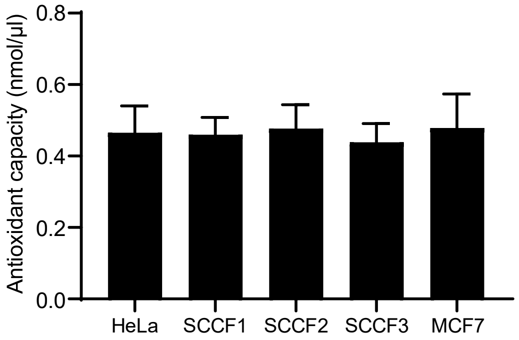

Figure S4. Antioxidant capacity of several cell lines. Differences in the capacity of the panel of cell lines to deal with reactive oxygen species were investigated by disrupting the cells and measuring the total antioxidant capacity in these. The graph displays the concentration of antioxidant per 200,000 cells for each cell line.

References

1. Oliveira S, Van Dongen G a MS, Stigter-Van Walsum M, et al. Rapid visualization of human tumor xenografts through optical imaging with a near-infrared fluorescent anti-epidermal growth factor receptor nanobody. Mol Imaging. 2012; 11: 33–46.

2. Kaplan M, Narasimhan S, de Heus C, et al. EGFR Dynamics Change during Activation in Native Membranes as Revealed by NMR. Cell. 2016; 167: 1241-1251.e11.

3. Mamot C, Drummond DC, Greiser U, et al. Epidermal growth factor receptor (EGFR)-targeted immunoliposomes mediate specific and efficient drug delivery to EGFR- and EGFRvIII-overexpressing tumor cells. Cancer Res. 2003; 63: 3154–3161.

4. Heukers R, van Bergen en Henegouwen PMP, Oliveira S. Nanobody–photosensitizer conjugates for targeted photodynamic therapy. Nanomedicine Nanotechnology, Biol Med. 2014; 10: 1441–1451.
